# 

**Published:** 1906-08-04

**Authors:** 


CONTENTS.

**Smell in Health and Disease** - - - - - 307
**Psychic Effects of the San Francisco Earthquake** 308
**Annotations** - - - - - - - - - - - 309
**Medical Opinion and Movement** - - - - - - 310
**Hospital Clinics—**
A Clinical Lecture on Infantile Paralysis - - - - - 311
The Treatment of Diphtheria - - - - - - - 313
**Practical Notes on Diagnosis and Treatment** - 315
**Special Analytical and Health Commission on Tinned and Preserved Food** - - - - - 316
**Remedies and their Uses** - - - - - - - 317
**New Appliances and Things Medical** - - - - 318
**The Book World of Medicine and Science** - - 318
**Medical Vacancies** - - - - - - - - - 318
**Hospital Administration—**
Current Hospital Topics - - - - - - - - 319
British and American Hospitals - - - - - - - 320
Social and Poor Law Problems - - - - - - - 322
**Editor's Letter-Box** - - - - - - - - - 322

*NURSING SECTION.*

**Notes on News from the Nursing World—** Midwifery Training in Poor-law Infirmaries—Nurses and Evidence—Keeping the Staff at a Minimum—An Inquiry at Hope Hospital—Holidays at the Cost of Extra Duty—Military Nursing Service—Important Appointments to the South-Eastern Fever Hospital—Swimming Competitions by Nurses—The Nurses' Union—Cumberland Infirmary—Lady Dufferin on District Nursing—The New Home at Reading—Asleep on Duty—A Vacancy that was not Filled—A Step in the Right Direction—Seaside and Holiday Apartments—A Twenty-Thousand Shilling Fund—Short Items - - - - - - - 253-256
**The Nursing Outlook** - - - - - - - 257
**The Care and Nursing of the Insane** - - - - - 258
**The Nurse's Clinic** - - - - - - - - - 259
**Incidents in a Nurse's Life** - - - - - - - 260
**A Visit to a Dutch Hospital** - - - - - - 261
**Central Midwives Board** - - - - - - - 261
**Queen Victoria's Jubilee Institute for Nurses** - 262
**State Registration** - - - - - - - - - 262
**Appointments** - - - - - - - - - - 263
**Everybody's Opinion** - - - - - - - - 263
**Presentations** - - - - - - - - - - 264
**The Nurses' Bookshelf** - - - - - - - - 264
**A Book and Its Story** - - - - - - - - 265
**Notes and Queries** - - - - - - - - - 266
**For Reading to the Sick** - - - - - - - 266

